# Momentum-selective orbital hybridisation

**DOI:** 10.1038/s41467-022-32643-z

**Published:** 2022-09-02

**Authors:** Xiaosheng Yang, Matteo Jugovac, Giovanni Zamborlini, Vitaliy Feyer, Georg Koller, Peter Puschnig, Serguei Soubatch, Michael G. Ramsey, F. Stefan Tautz

**Affiliations:** 1 Peter Grünberg Institut (PGI-3), Forschungszentrum Jülich, 52425 Jülich, Germany; 2 Jülich Aachen Research Alliance (JARA)–Fundamentals of Future Information Technology, 52425 Jülich, Germany; 3 Experimental Physics IV A, RWTH Aachen University, 52074 Aachen, Germany; 4 Peter Grünberg Institut (PGI-6), Forschungszentrum Jülich, 52425 Jülich, Germany; 5 Faculty of Physics and Center for Nanointegration Duisburg-Essen (CENIDE), Universität Duisburg-Essen, 47047 Duisburg, Germany; 6 Institute of Physics, University of Graz, NAWI Graz, 8010 Graz, Austria; 7 Present Address: Elettra - Sincrotrone Trieste, S.S. 14 km 163.5, Basovizza, 34149 Trieste, Italy; 8 Present Address: Department of Physics, TU Dortmund University, Dortmund, Germany

**Keywords:** Surfaces, interfaces and thin films, Surfaces, interfaces and thin films, Chemical physics

## Abstract

When a molecule interacts chemically with a metal surface, the orbitals of the molecule hybridise with metal states to form the new eigenstates of the coupled system. Spatial overlap and energy matching are determining parameters of the hybridisation. However, since every molecular orbital does not only have a characteristic spatial shape, but also a specific momentum distribution, one may additionally expect a momentum matching condition; after all, each hybridising wave function of the metal has a defined wave vector, too. Here, we report photoemission orbital tomography measurements of hybrid orbitals that emerge from molecular orbitals at a molecule-on-metal interface. We find that in the hybrid orbitals only those partial waves of the original orbital survive which match the metal band structure. Moreover, we find that the conversion of the metal’s surface state into a hybrid interface state is also governed by momentum matching constraints. Our experiments demonstrate the possibility to measure hybridisation momentum-selectively, thereby enabling deep insights into the complicated interplay of bulk states, surface states, and molecular orbitals in the formation of the electronic interface structure at molecule-on-metal hybrid interfaces.

## Abstract (web summary)

When a molecule interacts chemically with a metal, its orbitals hybridise with metal states to form the new eigenstates of the coupled system. Here, the authors show that in addition to overlap in real space and energy, hybridizing states must fulfil a momentum-matching condition.

## Introduction

In molecular orbital (MO) theory^1^, the linear combination of atomic orbitals is the decisive element of chemical bonding^2^, while in the valence bond (VB) picture, it is the overlap between hybridised atomic valence orbitals^3^. In both models, if two atoms form a bond, the bonding strength is determined by the orbital overlap in the interaction potential^4,5^. This intuitive real-space picture of bonding is challenged if an atom bonds to the surface of a metal. The single overlap integral of the atom-atom bond must then be replaced by a **k**-dependent hybridisation *V*_**k**_, where the **k** denote the wave vectors of the fully delocalised metal wave functions^6–8^. Many different wave functions with distinct **k** will potentially overlap and hybridise with the local atomic states at least to some extent. While this complex interaction can be unravelled with the help of ab initio calculations, because they routinely afford a view on individual wave functions, a differentiated **k**-resolved analysis of chemical bonding has so far not been accessible in experiment, despite first hints that momentum matching may play a crucial role^9^.

**Fig. 1:**
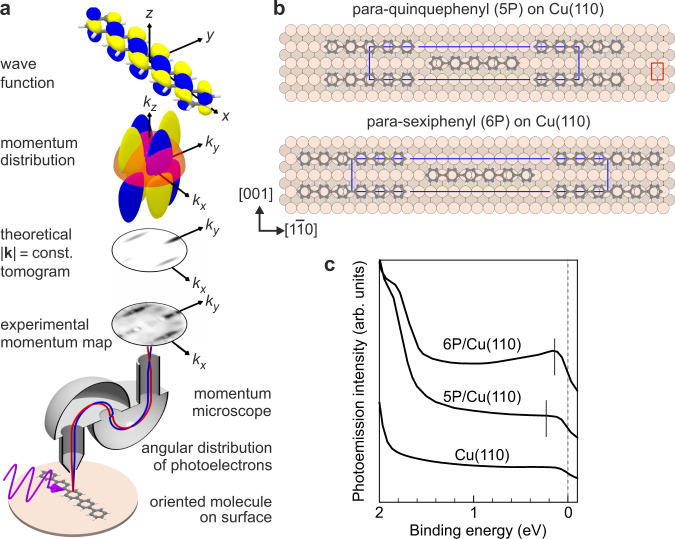
Photoemission orbital tomography. **a** Incoming photons (purple) excite photoelectrons from an ordered molecular layer. Their angular distribution is collected by the momentum microscope, which here is composed of two hemispherical electron analysers. The momentum microscope yields an energy-filtered two-dimensional **k**_∥_-space image of the photoemission intensity. This experimental momentum map corresponds to tomograms of the orbital's momentum distribution, obtained by a hemispherical cut (Ewald sphere) through the three-dimensional Fourier transform of the orbital's wave function. The size of the Ewald sphere is determined by the kinetic energy of the photoelectrons. **b** Structural models of ordered monolayers of 5P and 6P on Cu(110). Red and blue rectangles mark the unit cells of, respectively, metal surface and molecular monolayers. **c** Photoemission spectra of clean Cu(110), 5P/Cu(110), and 6P/Cu(110). Vertical solid lines mark the binding energies at which the corresponding momentum maps in Fig. 2b and Supplementary Fig. 2b were recorded. The vertical dashed line marks the Fermi level.

Here, we present a detailed experimental view of hybrid orbitals at a molecule-on-metal interface. The experiments are based on photoemission orbital tomography (POT)^10–17^. In POT, one collects photoelectrons emitted from an orbital in the full half-space above the sample and records their angular distribution. This leads to so-called momentum maps, which are tomograms of the momentum distribution of the orbital (Fig. 1a). As we show in this paper, the momentum distribution of an orbital may be profoundly affected by chemical bonding. This allowed us to trace hybridisation in a **k**-resolved (i.e., wave-function-resolved) manner, giving remarkable insights into the interaction at molecule-on-metal interfaces. For example, we found that in the hybridisation process partial waves that do not fulfil a **k**-matching condition are quenched from the orbital’s original momentum distribution.

## Results

### Momentum maps

For our experiments, we grew monolayers of two oligophenyls, para-quinquephenyl (5P) and para-sexiphenyl (6P) on Cu(110) (Fig. 1b). Both form similar superstructures with centred unit cells, c(18 × 2) and c(22 × 2)^18^, respectively (Supplementary Fig. 1). A structural analysis suggests that the molecules are oriented along the [1$\overline{1}$0] direction on Cu(110) (Fig. 1b), which is confirmed by POT. POT experiments were carried out with the NanoESCA photoemission electron microscope at Elettra Sincrotrone Trieste, Italy^19^.

**Fig. 2:**
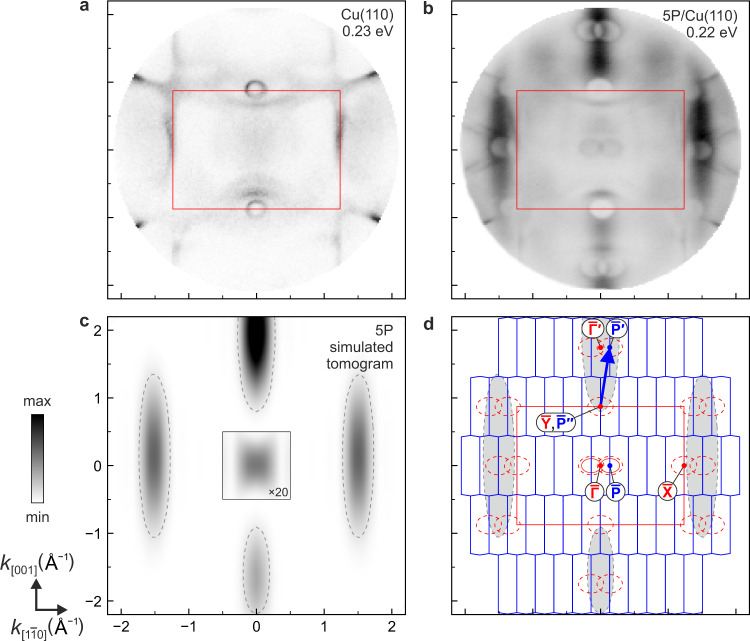
Momentum maps of clean Cu(110) and para-quinquephenyl molecules (5P) on Cu(110). **a** Experimental momentum map of clean Cu(110) at 0.23 eV binding energy. **b** Experimental momentum map of 5P/Cu(110) at 0.22 eV binding energy. The experimental momentum maps in panels a and b correspond to **k**_∥_-resolved densities of states. **c** Theoretically simulated tomogram of free planar 5P. Dashed lines mark the molecular emission lobes. Within the marked box centred at **k**_∥_ = 0, the intensity is increased by a factor of 20. **d** Schematic drawing illustrating the observed features in the experimental momentum map. The red rectangle shows the 1BZ of the Cu(110) surface, with high-symmetry points labeled in red. The blue polygons represent the periodically repeated first Brillouin zones of the ordered 5P/Cu(110) overlayer structure, with high-symmetry points labeled in blue. A representative reciprocal lattice vector is shown by the blue arrow. Grey dashed lines (from panel **c**) and grey shaded areas mark the molecular emission lobes. The red dashed ellipses mark the surface-projected bulk band gap at $\overline{{{{{{{{\rm{Y}}}}}}}}}$ and its observed replica, while the two solid red ellipses around $\overline{{{{{{{{\rm{P}}}}}}}}}$ points close to $\overline{{{\Gamma }}}$ mark the molecule-on-metal hybrid interface state (see text for more details).

In Fig. 2a, the momentum map of the clean Cu(110) surface is displayed. It was measured at a binding energy of 0.23 eV below the chemical potential. Most notably, we observed an elliptical feature that originates from the well-known Shockley surface state at the $\overline{{{{{{{{\rm{Y}}}}}}}}}$ point of the first surface Brillouin zone (1BZ) of Cu(110)^20,21^. In addition, several sharp sp bands are visible in Fig. 2a. Turning to 5P/Cu(110), we recorded a momentum map (Fig. 2b) with a rich structure at a binding energy where the momentum-integrated spectrum in Fig. 1c exhibits weak molecule-induced features: while the sharp substrate bands of Fig. 2a are blurred and reduced in intensity, the momentum map is dominated by two pairs of cigar-like lobes at **k**_∥_ ≈ ( ± 1.5, 0) Å^−1^ and (0, ± 1.9) Å^−1^. Inspecting the simulated tomograms of the lowest unoccupied molecular orbital (LUMO) of free 5P at the corresponding kinetic energy in Fig. 2c, we see that the emission lobes in Fig. 2b derive from 5P’s LUMO. This indicates a charge transfer from the metal to the molecule that leads to a partially filled LUMO, from which photoemission became possible. The positions of the lobes in the LUMO momentum map of 5P are inherited from the LUMO of benzene and are therefore essentially the same for all linear oligophenyls^10,22^. The widths of the lobes in ${k}_{[1\bar{1}0]}$ direction are reciprocally related to the number of phenyl units^10^, while the extensions of the lobes along *k*_[001]_ indicate the widths of the molecules. We note that in the *xy* plane the LUMO of 5P is a totally symmetric orbital (Fig. 1a). As such, it also has a minor lobe centred at **k**_∥_ = 0.

### Scattering of interface electrons by the molecular overlayer

Beside the molecular lobes, the 5P/Cu(110) momentum map in Fig. 2b shows a wealth of additional features: at the $\overline{{{{{{{{\rm{Y}}}}}}}}}$ points and several other **k**_∥_ positions, we discern elliptical intensity depletions at which the intensity of the LUMO lobes becomes extinct. If two depletions overlap, as is the case at the $\overline{{{\Gamma }}}^{\prime}$ point, a thin line of intensity occurs just outside each depletion zone. Finally, the elliptical surface state at $\overline{{{{{{{{\rm{Y}}}}}}}}}$ that is clearly seen for Cu(110) is apparently destroyed on adsorption of the molecules, while two elliptical rings of approximately the same size have appeared close to the $\overline{{{\Gamma }}}$ point. The most notable features of the 5P/Cu(110) momentum map are schematically summarised in Fig. 2d in red. The figure also shows the periodically repeated 1BZ of the molecular superstructure (blue) with special points $\overline{{{{{{{{\rm{P}}}}}}}}}$. This illustrates that all of the observed elliptical depletion zones can be replicated from the ones at the $\overline{{{{{{{{\rm{Y}}}}}}}}}$ point by reciprocal lattice vectors of the molecular superstructure via zone folding. The same is true for the elliptical rings at the $\overline{{{{{{{{\rm{P}}}}}}}}}$ points close to $\overline{{{\Gamma }}}$: these appear to be replications of the original (but apparently destroyed) surface state at $\overline{{{{{{{{\rm{Y}}}}}}}}}$. The momentum map of 6P/Cu(110) is displayed in the supplement (Supplementary Fig. 2). It is completely analogous to the one of 5P/Cu(110) in Fig. 2, proving the generic character of the observed phenomenology.

**Fig. 3:**
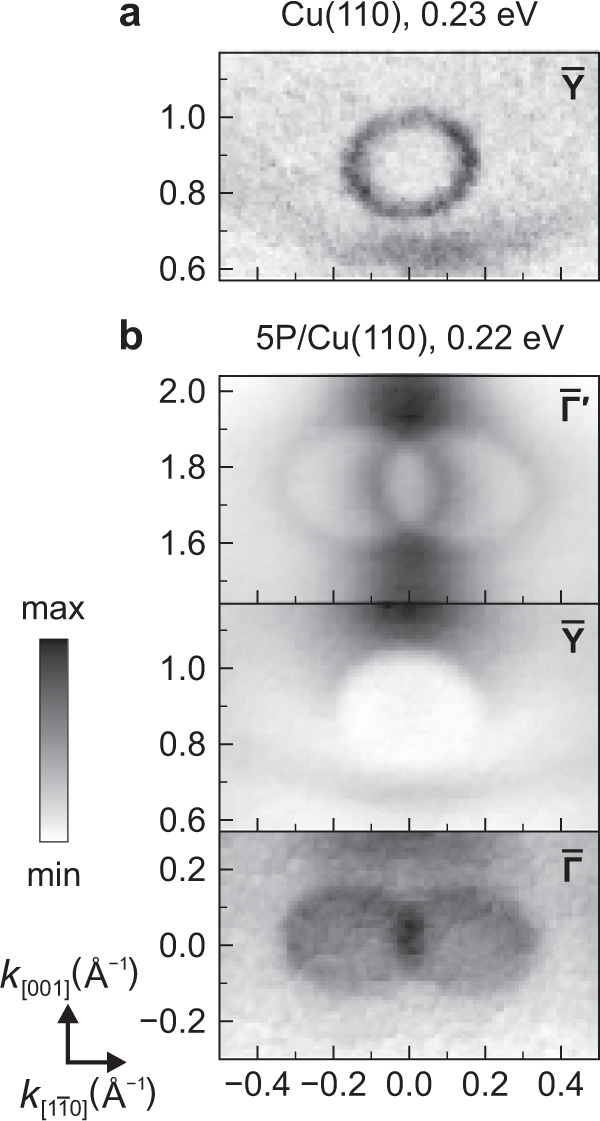
Zoomed momentum maps around high-symmetry points of Cu(110). **a** Clean Cu(110) around $\overline{{{{{{{{\rm{Y}}}}}}}}}$. **b** 5P/Cu(110) around ${\overline{{{\Gamma }}}}^{\prime}$, $\overline{{{{{{{{\rm{Y}}}}}}}}}$, and $\overline{{{\Gamma }}}$. The different sizes of the depletion zones at $\overline{{{{{{{{\rm{Y}}}}}}}}}$ and close to ${\overline{{{\Gamma }}}}^{\prime}$ on the one hand and the interface state close to $\overline{{{\Gamma }}}$ on the other hand can be observed clearly.

The first question that arises addresses the nature of the depletion zones. Their magnified views in Fig. 3 (for 6P/Cu(110) cf. Supplementary Fig. 3) at $\overline{{{{{{{{\rm{Y}}}}}}}}}$ and close to $\overline{{{\Gamma }}}^{\prime}$ show that they are larger than the elliptical surface state of Cu(110). This visual impression is confirmed by a combined analysis of momentum maps and band maps in Supplementary Fig. 4 (for 6P/Cu(110) cf. Supplementary Fig. 5), in which we find that the depletion zones at $\overline{{{{{{{{\rm{Y}}}}}}}}}$ correspond to a parabola with its apex at 594 ± 25 meV and with an effective mass of 0.44  ± 0.05 *m*_*e*_ in the ${k}_{[1\bar{1}0]}$ direction (the corresponding value in *k*_[001]_ direction is 0.29  ± 0.03 *m*_*e*_), in excellent agreement with the literature value for the lower edge of the surface-projected bulk band gap of Cu(110) (0.42 *m*_*e*_ in ${k}_{[1\bar{1}0]}$ direction)^23^, but significantly larger than for the Cu(110) surface state at $\overline{{{{{{{{\rm{Y}}}}}}}}}$ itself (Supplementary Fig. 6) for which we find an apex at 473 ± 13 meV and effective masses 0.34  ± 0.04 *m*_*e*_ and 0.23  ± 0.03 *m*_*e*_ in ${k}_{[1\bar{1}0]}$ and *k*_[001]_ directions, respectively, in very good correspondence with the literature values^23^. Precisely the same values (within experimental accuracy) as at $\overline{{{{{{{{\rm{Y}}}}}}}}}$ are found for the depletion zones at $\overline{{{{{{{{\rm{P}}}}}}}}}^{\prime}$ close to $\overline{{{\Gamma }}}^{\prime}$. We therefore conclude that the depletion zones at $\overline{{{{{{{{\rm{Y}}}}}}}}}$ and $\overline{{{{{{{{\rm{P}}}}}}}}}^{\prime}$ originate from the well-known band gap in the surface-projected bulk band structure at the $\overline{{{{{{{{\rm{Y}}}}}}}}}$ point of (110) surfaces of copper and other noble metals, in which their Shockley surface states reside^24–26^.

The observation of the surface-projected bulk band gaps in the momentum map of Fig. 2b is notable for two reasons: First, the replication of substrate band structure features such as the $\overline{{{{{{{{\rm{Y}}}}}}}}}$-point gap reveals that within the escape depth of the photoelectrons, the 5P molecules modulate the potential felt by the copper substrate electrons, thus creating a new, folded band structure. Because the phenyl rings constitute the elementary structural units of 5P, we expect that their oligomeric (i.e. locally periodic) arrangement dominates the modulation of the potential in which the substrate electrons are Bragg-scattered. This is exactly what we observe: since the size of the phenyl rings approximately matches the surface unit cell of Cu(110), we only observe replicas close to the Cu(110) 1BZ zone boundary. In contrast, in the *k*_[001]_ direction the bulk band gap is replicated at all reciprocal lattice vectors, because in this direction the primitive unit cell of the overlayer has the same extension as the Cu(110) unit cell.

### Experimental k_∥_-resolved density of states

Second, the observation of the band gaps in the momentum map is remarkable because band gaps in the original band structure become filled when the surface-projected bulk band structure of the Cu(110) surface is folded into the smaller 1BZ of the molecular superstructure. So, why does one still observe signs of the $\overline{{{{{{{{\rm{Y}}}}}}}}}$-point gap in the momentum map, not only at the original but also at zone-folded positions? This can be understood by referring to the equation that describes the information content of the momentum map according to the one-step model of the photoemission process in the plane-wave approximation for the final state,

1 $$I({E}_{b},{{{{{{{{\bf{k}}}}}}}}}_{\parallel}; h \nu,{{{{{{{\bf{A}}}}}}}})\propto \mathop{\sum }\limits_{n}^{{{{{{{{\rm{occ}}}}}}}}}\mathop{\sum }\limits_{{{{{{{{\bf{q}}}}}}}}}^{{{{{{{{\rm{1BZ}}}}}}}}}|{{{{{{{\bf{A}}}}}}}}\cdot {{{{{{{\bf{k}}}}}}}}{|}^{2}|\langle {e}^{i{{{{{{{\bf{k}}}}}}}}\cdot {{{{{{{\bf{r}}}}}}}}}|{\psi}_{n,{{{{{{{\bf{q}}}}}}}}}\rangle {\vert }^{2}\\ \times \delta ({\epsilon}_{n,{{{{{{{\bf{q}}}}}}}}}+{{\Phi }}+{E}_{{{{{{{\rm{{kin}}}}}}}}} - h \nu),$$

where *E*_*b*_ ≡ *hν* − Φ − *E*_kin_, Φ is the work function, $|{{{{{{{\bf{k}}}}}}}} \vert=\sqrt{2m{E}_{{{\mathrm{kin}}}}/{\hslash }^{2}}$, and the sums are carried out over all occupied states *n*, **q**, where *n* is a band index and **q** the wave vector in the 1BZ of the molecular superstructure^27^. $\mathop{\sum }\nolimits_{n}^{{{{{{{{\rm{occ}}}}}}}}}\mathop{\sum }\nolimits_{{{{{{{{\bf{q}}}}}}}}}^{{{{{{{{\rm{1BZ}}}}}}}}}\delta ({\epsilon }_{n,{{{{{{{\bf{q}}}}}}}}}-{E}_{b})$ is the density of states at *E*_*b*_ of the combined molecule-on-metal system with energy levels *ϵ*_*n*,**q**_, while the matrix elements 〈*e*^*i***k**⋅**r**^∣*ψ*_*n*,**q**_〉 project the wave functions *ψ*_*n*,**q**_ of the combined system onto partial waves with definite **k**. Note that for given *E*_*b*_ and **k**_∥_, *k*_*z*_ is fixed by energy conservation and the parameters Φ and *hν*. The matrix elements in Eq. (1) thus weight the contribution of each state vector *ψ*_*n*,**q**_ to the total density of states at *E*_*b*_ according to its **k**_∥_ content. Apart from an additional polarisation factor ∣**A** ⋅ **k**∣^2^ that stems from the photoemission process, the momentum maps of photoemission orbital tomography thus provide a **k**_∥_-resolved density of states of the system under study. The polarisation factor only leads to a weak asymmetry in the measured momentum maps that can be ignored. We note that while the definition of a **k**_∥_-resolved density of states is generic, the fact that we can measure it by photoemission orbital tomography is contingent upon the validity of the plane-wave final state approximation, which has been solidly confirmed for systems of the kind studied here^15^ and is again confirmed in the present work, as we will see below. In this approximation, the photoemission process actually ‘unfolds’ the density of states into contributions from the partial waves of the underlying wave functions. It does this because the partial wave *e*^*i***k**⋅**r**^ in Eq. (1) enters this equation as the (plane-wave) final state of photoemission process. Thus, the conceptual projection onto partial waves in the **k**_∥_-resolved density of states is executed experimentally by the projection on the plane-wave final state wave function of the photoemission process.

In the light of Eq. (1), we can finally interpret the depletion zones in Fig. 2b as minima in the **k**_∥_-resolved density of states of the interface 5P/Cu(110). From their locations in all those places to which the $\overline{{{{{{{{\rm{Y}}}}}}}}}$ point is zone-folded by the molecular overlayer structure, we can conclude that these minima derive from the $\overline{{{{{{{{\rm{Y}}}}}}}}}$ point band gap in the surface-projected bulk band structure of the bare Cu(110) surface. The gaps, although filled in the band structure, apparently become visible again through the very unfolding that is implicit in the photoemission process.

### Momentum matching in the hybridisation of molecular orbitals with bulk metal states

The interpretation of the momentum maps as **k**_∥_-resolved density of states opens the door to the analysis of momentum-selective hybridisation. Indeed, the Bragg scattering of the bulk metal states at the 5P/Cu(110) interface by the molecular lattice does not convey the complete picture – there is also hybridisation between the metal and the molecules. Comparing the **k**_∥_-resolved density of states of the molecule-on-metal hybrid states at the 5P/Cu(110) interface in Fig. 2b to the Ewald projection of the three-dimensional Fourier transform^15^ of the LUMO in Fig. 2c, which in the above logic is nothing but the **k**_∥_-resolved density of states of the molecule at the binding energy of this orbital, we see that at the interface certain partial waves of the LUMO are missing: whenever partial waves of the LUMO coincide with the gap, they become extinct – at these **k**_∥_, there are no hybrid states at the 5P/Cu(110) interface. An intuitive conceptual explanation of this behaviour is that the hybridising orbital of the adsorbing molecule decomposes into its **k**_∥_ partial waves before the hybridisation with metal states, which also have a definite **k**_∥_, proceeds. While partial waves of the LUMO and of the substrate with matching wave vectors may hybridise without any problem (if they overlap in space and energy), all partial waves of the LUMO which do not have a counterpart with the same **k**_∥_ among the metal states actually become extinct. The extinction effectively adjusts the momentum distribution of the LUMO to the one of the metal surface. In conclusion, our data thus show that hybridisation does not only require coincidence of the hybridising states in energy and real space, but also in momentum space. Through this momentum matching condition it is also safeguarded that each hybrid state has a unique **k**_∥_ quantum number, as it must at periodic interfaces such as 5P/Cu(110).

Up to this point, we have interpreted the momentum maps with all sub-structures, including the extinctions in specific **k**_∥_ regions, to reflect the initial state wave functions of the photoemission process from which the momentum maps emerge, i.e. the molecule-on-metal hybrid states at the interface. To substantiate this interpretation and in particular to rule out a possible impact of photoemission final state scattering, we performed ab-initio electronic structure calculations in the framework of density functional theory (DFT) for the 5P/Cu(110) interface and simulated the momentum maps as tomograms according to Eq. (1) (cf. Supplementary Movie 1). To correctly account for the Shockley surface state, which seems to play a role in the experimental momentum map of Fig. 2b, we modelled the Cu(110) surface within a repeated-slab approach, each slab consisting of 25 layers of copper. Such a large number of metal layers prevents cross-talk between surface states formed at the top and the bottom of the slab and has been shown to yield converged wave functions of the $\overline{{{{{{{{\rm{Y}}}}}}}}}$ point surface state^28^. In our simulation of momentum maps, we used a damped plane wave as the final state to mimic the surface sensitivity of ultraviolet photoemission^27^. This means that in Eq. (1) we in fact replaced *e*^*i***k**⋅**r**^ inside the substrate (i.e. for *z* < *z*_0_) by ${e}^{i{{{{{{{\bf{k}}}}}}}}\cdot {{{{{{{\bf{r}}}}}}}}+\gamma (z-{z}_{0})}$. With the exception that thereby we restricted both the photoemission process and the **k**_∥_-resolved density of states to the interface region in which hybridisation occurs, this does not change any of the arguments given above. Note that we also applied a dense sampling of the Brillouin zone to ensure the required **k**_∥_ resolution in the simulated tomograms as detailed in the Methods Section.

**Fig. 4:**
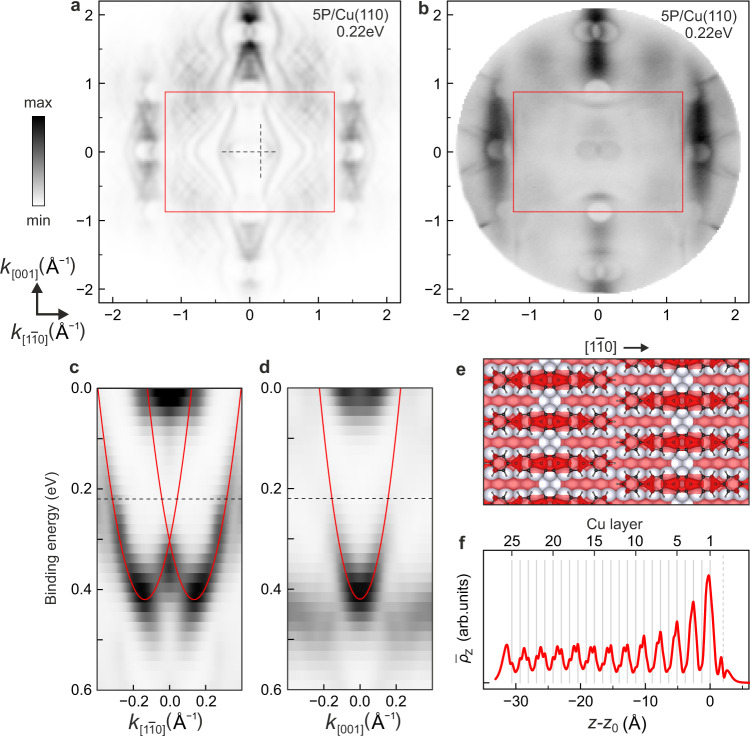
Comparison of simulated and experimental momentum maps of 5P/Cu(110). **a** Theoretical momentum map, based on a DFT calculation for the 5P/Cu(110) interface and simulated as a tomogram according to Eq. (1), but with an exponentially damped plane wave as final state. **b** Measured momentum map of 5P/Cu(110), reproduced from Fig. 2b for ease of comparison with panel **a**. The red rectangles in panels **a** and **b** show the 1BZ of the Cu(110) surface. **c**, **d** Simulated band maps along two high-symmetry directions of Cu(110), plotted for **k**_∥_ values along the horizontal and vertical dashed lines in panel a. Red solid lines mark the parabolically dispersing 5P/Cu(110) interface state. **e** Top view of the electron density distribution of the 5P/Cu(110) interface state at $\overline{{{{{{{{\rm{P}}}}}}}}}$, superimposed on the atomic model of the 5P/Cu(110) interface. **f** Side view of the same electron density, but plane-averaged parallel to the slab surfaces and plotted across the slab that was used for the 5P/Cu(110) calculations. Solid and dashed grey lines denote the positions of atomic layers of copper and the molecular plane of 5P, respectively.

Our DFT calculations indeed confirmed that the 5P LUMO becomes fractionally charged upon adsorption. Significantly, all salient features of the experimental momentum maps are also present in the simulated tomogram displayed in Fig. 4a (for ease of comparison, the experimental momentum map of Fig. 2b has been reproduced as Fig. 4b next to the simulated tomogram). This includes the gap replications in the LUMO’s emission lobes and the ellipses that have appeared at the $\overline{{{{{{{{\rm{P}}}}}}}}}$ points close to $\overline{{{\Gamma }}}$. Even the diffuse background intensity modulation between the major molecular lobes appears to be captured in the simulation, albeit with an additional fine structure that arises from the finite number of copper layers in the calculation. Importantly, since the simulation assumed a damped plane-wave final state, it does not contain the effects of scattering of the outgoing photoelectron. Hence, the remarkable agreement between experiment and simulation (compare Figs. 4a with 2b/4b) precludes that the observed structures in the momentum maps are due to final-state scattering effects. This conclusively confirms our interpretation that a momentum matching condition is active during hybridisation at the 5P/Cu(110) molecule-on-metal interface.

### Momentum matching and the formation of a hybrid interface state

The simulation also allows an analysis of the fate of the Cu(110) surface state upon adsorption of the molecular layer. The influence of molecular layers on Shockley surface states of metals has long been subject to intense research^29–33^. While the common understanding is that such surface states are either partially^34–38^ or completely^39,40^ depopulated on adsorption, because they tend to be shifted up in energy, in some notable cases it was shown that they hybridise with specific molecular orbitals to form a hybrid interface state^29,41,42^, while in other cases they coexist apparently unaffected with even a full adsorbate layer^43^. The reason for this seemingly disparate behaviour is currently not understood. Therefore, extending the above momentum-resolved analysis of hybridisation at molecule-on-metal interfaces from bulk metal to surface states may hold the key to a more complete understanding of the interaction between surface states and molecular adsorbates, as well as the conditions under which genuine hybrid interface states may emerge.

Experiments suggest that the Brillouin zone folding caused by the overlayer structure plays a role for the surface state, too: at the $\overline{{{{{{{{\rm{P}}}}}}}}}$ points close to $\overline{{{\Gamma }}}$ we find photoemission intensity distributed in two elliptical rings that appear notably smaller than the depletion zones at $\overline{{{{{{{{\rm{Y}}}}}}}}}$ and $\overline{{{{{{{{\rm{P}}}}}}}}}^{\prime}$ (Figs. 2b/4b). This can also be seen in the band maps of Supplementary Fig. 4, where the observed photoemission intensity appears within the surface-projected bulk band gap, roughly aligned with corresponding states that come into view in the simulation (Fig. 4a,c,d). These calculated states for the 5P/Cu(110) interface exhibit a parabolic dispersion with effective masses approximately twice as large as that of the calculated Cu(110) surface state [0.61 *m*_*e*_ (0.46 *m*_*e*_) in ${k}_{[1\bar{1}0]}$ (*k*_[001]_) direction for 5P/Cu(110) vs. 0.30 *m*_*e*_ (0.23 *m*_*e*_) for Cu(110)]. However, it is not uncommon that molecular overlayers modify the effective mass of a Shockley surface state^33^. Moreover, we observe only a small binding energy shift of ~ 0.1 eV between the apices of the calculated Cu(110) surface state at the $\overline{{{{{{{{\rm{Y}}}}}}}}}$ (Supplementary Fig. 7c,d) and the corresponding 5P/Cu(110) states at the $\overline{{{{{{{{\rm{P}}}}}}}}}$ points (Fig. 4c,d). We note in passing that the increased effective mass of the latter state in the calculation fortuitously compensates its binding energy shift, such that in the calculated momentum map in Fig. 4a the state at $\overline{{{{{{{{\rm{P}}}}}}}}}$ appears to have approximately the same size as the original surface state at $\overline{{{{{{{{\rm{Y}}}}}}}}}$ in Supplementary Fig. 7a. Taking all the evidence together, experimental data for 5P/Cu(110) strongly suggest that at the $\overline{{{{{{{{\rm{P}}}}}}}}}$ points on either side of $\overline{{{\Gamma }}}$ we detect descendants of the Cu(110) surface state. In contrast, at $\overline{{{{{{{{\rm{P}}}}}}}}}^{\prime\prime}$ (which coincides with $\overline{{{{{{{{\rm{Y}}}}}}}}}$) we find no such state in experiment, and only a very weak one in the simulation (Supplementary Fig. 8). And at the $\overline{{{{{{{{\rm{P}}}}}}}}}^{\prime}$ points close to $\overline{{{\Gamma }}}^{\prime}$ no indications of such a state can be discerned at all.

That the states at $\overline{{{{{{{{\rm{P}}}}}}}}}$ in Fig. 2b/4b are indeed descendants of the original surface state at the $\overline{{{{{{{{\rm{Y}}}}}}}}}$ point of Cu(110) can finally be concluded by analysing the simulated real-space electron densities associated with the bottom of the paraboloids. As shown in Supplementary Fig. 7e, the electron density of the surface state is found between the atomic rows of the Cu(110) surface, but with alternating signs of the wave function such that it appears at the $\overline{{{{{{{{\rm{Y}}}}}}}}}$ point and not at $\overline{{{\Gamma }}}$. After molecular adsorption, the electron density still resides between the copper rows. However, the modulation of the surface potential has led to it being now concentrated under the molecules (Fig. 4e). This adjustment of its periodicity to the overlayer moves the state into the vicinity of the $\overline{{{\Gamma }}}$ point; the offset to $\overline{{{{{{{{\rm{P}}}}}}}}}$ is a direct consequence of the fact that the rows of molecules are staggered.

Why, however, are the descendant states not seen at the $\overline{{{{{{{{\rm{P}}}}}}}}}^{\prime}$ and $\overline{{{{{{{{\rm{P}}}}}}}}}^{\prime\prime}$ points which are equivalent to $\overline{{{{{{{{\rm{P}}}}}}}}}$? As we will show now, this can be rationalised as a consequence of momentum matching during the hybridisation of the Cu(110) surface state with the 5P LUMO, whereby a 5P/Cu(110) hybrid interface state is formed. Because the Bragg scattering by the overlayer concentrates the former surface state under the molecule, and its energy moreover coincides with that of the LUMO (pulled below the chemical potential upon adsorption), this hybridisation is structurally and energetically favoured. Momentum matching is then acting as the third determinant which governs the conversion of the surface state into a hybrid interface state: as a totally symmetric orbital in the *xy* plane, the 5P LUMO has a minor lobe centred at the $\overline{{{\Gamma }}}$ point (see Fig. 2c); there, the surface state and the LUMO are thus momentum matched and therefore can hybridise. On the other hand, we recall that – due to the constraint of momentum matching between the LUMO and bulk metal states – the partial waves of the LUMO around $\overline{{{{{{{{\rm{P}}}}}}}}}^{\prime}$ and $\overline{{{{{{{{\rm{P}}}}}}}}}^{\prime\prime}$ do not survive the hybridisation with Cu(110) surface. Therefore, they are not any more available for a hybridisation with the surface state, and this in turn means that corresponding momentum components of the surface state do not prevail, although as Bragg-scattered replications they would be allowed by the translational symmetry of the molecular overlayer. Notably, the strong attenuation or extinction of the interface state at $\overline{{{{{{{{\rm{P}}}}}}}}}^{\prime}$ and $\overline{{{{{{{{\rm{P}}}}}}}}}^{\prime\prime}$ washes out the constrictions of its real-space wave function between the molecules which can be seen in Fig. 4e. Hence, in spite of the concentration of its wave function below the molecules by Bragg scattering, the interface state remains delocalised along the **k**_[001]_ direction.

The hybridisation of the surface state with the 5P LUMO and the concurrent formation of a hybrid interface state can clearly be discerned in its calculated wave function, which is plotted in Fig. 4f for **k**_∥_ = 0 ($\overline{{{\Gamma }}}$ point). It shows a double peak centred around the position of the molecule above the surface, originating from the LUMO orbital lobes above and below the plane of the molecule (in the plane of the molecule, the LUMO has a nodal plane). This proves that beyond the metal surface, the wave functions of the original Shockley surface state are continued by molecular states – clearly, this is only possible if states on both sides of the surface are momentum matched. We note that this continuation of the tail of the Shockley surface state by a molecular wave function is clearly associated with the concentration of the interface state wave function below the molecules as seen in Fig. 4e, and with a corresponding modulation of the surface potential. It may in fact also play a role in the charge transfer into the LUMO, in the sense that some of the transferred charge may indeed come from the original surface state.

A similar continuation of the Shockley wave function by molecular states has been reported for PTCDA/Ag(111)^29,41,42^. In that case, however, it is the LUMO+1, which hybridises (as a totally symmetric orbital in the *xy* plane, it also has a minor lobe at $\overline{{{\Gamma }}}$ where the surface state of the Ag(111) surface is located), because the surface state is shifted up by 0.6 eV into the unoccupied part of the spectrum^29,41,42^. For PTCDA/Ag(111), the lateral scattering potential that concentrates the interface state below the molecule was actually determined from the gaps that open at the 1BZ boundary by Bragg scattering^32^. The large binding energy shift of the interface state for PTCDA/Ag(111) with respect to the surface state of Ag(111) has been explained with a simple one-dimensional model for the potential outside the surface^31^. The molecular layer compresses this potential profile and thereby shifts the Shockley state up in energy. On the basis of this semiquantitative model, one would expect in the present case an upward shift of more than 1 to 1.5 eV, given the adsorption height of 2.1 to 2.2Å of 5P above Cu(110), even if it is taken into account that the low density of the adsorbate sensitively affects the upshift^33,44^. However, at (110) surfaces the surface state lives in the atomic trenches of the metal substrate (see the plot of the wave functions in Supplementary Fig. 7e) and, unlike for (111) surfaces, does not spill out far above the metal. In the trenches it is protected from the charge density of the approaching molecular layer, such that the potential felt by the Shockley surface state electrons will hardly be affected by the presence of the molecules. The simple model of ref. 31 therefore does not apply here. Indeed, our simulation predicts a rather small shift from 550 meV for the Cu(110) surface state to 420 meV for the 5P/Cu(110) hybrid interface state, cf. Fig. 4c-d and Supplementary Fig. 7c-d. The shift in experiment seems to be even smaller (from 473 ± 13 meV to ~ 420 meV) (Supplementary Figs. 4 and 6).

The interface state wave function in Fig. 4f reveals a very slow decay into the bulk that seems to saturate at about the 13th copper layer, in contrast to the surface state wave function displayed in Supplementary Fig. 7f. Following the standard classification, we should therefore more correctly refer to this state as an interface resonance. The saturation of the wave function decay clearly proves that metal states into which interface state electrons can transfer without changing their energy and their wave vector are available at $\overline{{{\Gamma }}}$, in full consistency with the fact that no minimum appears in the **k**_∥_-resolved density of states around $\overline{{{{{{{{\rm{P}}}}}}}}}$. This confirms our conjecture that bulk states exist close to $\overline{{{\Gamma }}}$ and therefore the minor **k**-space lobes of the LUMO do survive the process of hybridisation with the bulk states and therefore are able to continue the Shockley state into the molecular layer and the vacuum. This has to be contrasted with the situation at $\overline{{{{{{{{\rm{P}}}}}}}}}^{\prime}$ and $\overline{{{{{{{{\rm{P}}}}}}}}}^{\prime\prime}$, where according to the measured **k**_∥_-resolved density of states bulk states are essentially lacking, corresponding partial waves of the LUMO are destroyed, and the Shockley state cannot be continued into the molecular layer and the vacuum.

## Discussion

We presented a comprehensive momentum-resolved view of hybridisation at a highly ordered molecule-on-metal interface, based on the **k**_∥_-resolved density of states of the hybrid interface that is measured by photoemission orbital tomography within the plane-wave approximation for the final state of the photoemission process. In the present case of oligophenyls on the Cu(110) surface, the distinct momentum structure of the hybridising molecular orbital, as well as the existence of gaps in the surface-projected bulk band structure of the metal, allowed us to detect and trace in minute detail how hybrid orbitals are formed between the metal’s bulk states and the lowest unoccupied molecular orbital that becomes partially occupied in the process. Specifically, we could directly reveal momentum-space matching as an important governing principle of the hybridisation, next to spatial and energetic overlap and Bragg scattering of the interface electrons by the overlayer structure. Moreover, we could also observe how the surface state of the bare metal surface interacts and partially merges with these hybrid orbitals. Again, momentum matching turned out to be a governing principle. In summary, the momentum-space view afforded by our experiments has given us unprecedented insights into the complicated interplay of bulk states, surface states, and molecular orbitals in the formation of the electronic interface structure at molecule-on-metal hybrid interfaces. We anticipate that by spin detection in conjunction with photoemission orbital tomography, both spin-mixing and spin-conserving hybridisations, as were recently reported for inorganic materials^45^, will also become accessible in future. Furthermore, the **k**_∥_-resolved detection of hybridisation by photoemission orbital tomography may in future allow the recording of slow-motion videos of surface chemical reactions in which the formation of hybrid orbitals and chemical bonds can be traced in time with femtosecond resolution^17^.

## Methods

### Sample preparation

The sample preparation and the photoemission experiments were performed in ultra-high vacuum vessels (base pressures ~ 10^−10^ mbar). The Cu(110) substrate was cleaned by several cycles of sputtering with Ar^+^ ions (1.5 kV, 20 min per cycle) and annealing (800 K, 15 min per cycle). The cleanliness of the substrate surface was confirmed by low energy electron diffraction (LEED) and photoemission electron microscopy, in particular by the Cu(110) Shockley surface state that is known to be contamination-sensitive. Monolayers of 5P and 6P were deposited from a home-made Knudsen-type evaporator onto the Cu(110) surface held at room temperature. The structure of the molecular monolayers was controlled by LEED.

### Photoemission orbital tomography

The photoemission orbital tomography (POT) experiments were performed with the NanoESCA photoemission electron microscope^19^ (FOCUS GmbH/Omicron) at the NanoESCA beam line of the Elettra Sincrotrone Trieste, Italy. p-polarised light with a photon energy of 35 eV and an incidence angle of 65^∘^ with respect to the surface normal was used. During measurements, the sample was cooled with liquid nitrogen. The sample was oriented such that the plane of incidence included the [001] azimuth of Cu(110). The emitted photoelectrons were collected in the **k**_∥_ range from approximately − 2.0 to +2.0 Å^−1^. The kinetic energy of the photoelectrons was scanned with the photoemission electron microscope and a three-dimensional data cube of photoemission intensity *I*(*E*_kin_, *k*_*x*_, *k*_*y*_) was recorded. Momentum maps and band maps were obtained by taking sections of *I*(*E*_kin_, *k*_*x*_, *k*_*y*_) at fixed *E*_kin_ and **k**_∥_, respectively. To avoid beam damage due to the high-intensity focused photon beam, the sample position was laterally scanned during all measurements.

### Simulated tomograms

Ab initio electronic structure calculations are performed within the framework of density functional theory and utilising the VASP code^46–48^. The c(18 × 2) overlayer structure of 5P/Cu(110) determined from the experimental LEED data was used to set up a repeated slab model where the Cu substrate is modelled by 25 metallic layers. A vacuum layer of more than 25 Å was added between the periodic replica of the slabs. Additionally, a dipole layer was inserted in the vacuum region to avoid spurious electrical fields. A van der Waals-corrected^49^ generalised gradient approximation^50^ was employed for exchange-correlation effects, and the projector augmented wave (PAW)^51^ approach with a plane wave cutoff of 400 eV was utilised. The geometry optimisations were performed until all atomic forces are below 0.01 eVÅ^−1^, where we used a Monkhorst-Pack 8 × 2 × 1 grid of *k* points and a first-order Methfessel-Paxton smearing of 0.1 eV^52^. The wave functions for the relaxed 5P/Cu(110) interface served as initial states for the simulation of the photoemission tomograms, employing the one-step model of photoemission according to Eq. (1), but with a damped plane wave^27^ as final state (damping parameter of *γ* = 1.0 Å^−1^). With the increased 30 × 9 × 4 **k**_∥_-point sampling of the overlayer Brillouin zone, we achieved a **k**_∥_-resolution of 0.03 Å^−1^ and a binding energy resolution of 40 meV in the simulated tomograms.

## Supplementary information

Supplementary Information

Description of Additional Supplementary Files

Supplementary Movie 1

## Data Availability

The raw photoemission data used in this study are available in the Jülich DATA database under accession code 10.26165/JUELICH-DATA/6UG0DS.
