# Supplementary material for: Momentum-selective orbital hybridisation
Source: Nat Commun. 2022 Sep 2;13:5148. doi: 10.1038/s41467-022-32643-z (PMC9440066; doi:10.1038/s41467-022-32643-z)
Supplement: Supplementary file 1 — Supplementary Information [file 41467_2022_32643_MOESM1_ESM.pdf]

Supplementary Information
Momentum-selective orbital hybridisation

Xiaosheng Yang[1,2,3], Matteo Jugovac[4†], Giovanni Zamborlini[4‡], Vitaliy Feyer[4,5], Georg Koller[6], Peter Puschnig[6], Serguei Soubatch[1,2], Michael G. Ramsey[6*], and F. Stefan Tautz[1,2,3*]

[1]Peter Grünberg Institut (PGI-3), Forschungszentrum Jülich, 52425 Jülich, Germany
[2]Jülich Aachen Research Alliance (JARA)–Fundamentals of Future Information Technology, 52425 Jülich, Germany
[3]Experimental Physics IV A, RWTH Aachen University, 52074 Aachen, Germany
[4]Peter Grünberg Institut (PGI-6), Forschungszentrum Jülich, 52425 Jülich, Germany
[5]Faculty of Physics and Center for Nanointegration Duisburg-Essen (CENIDE), Universität Duisburg-Essen, 47047 Duisburg, Germany
[6]Institute of Physics, University of Graz, NAWI Graz, 8010 Graz, Austria
[†]present address: Elettra - Sincrotrone Trieste, S.S. 14 km 163.5, Basovizza, 34149 Trieste, Italy
[‡]present address: Department of Physics, TU Dortmund University, Dortmund, Germany
[*]email: michael.ramsey@uni-graz.at; s.tautz@fz-juelich.de

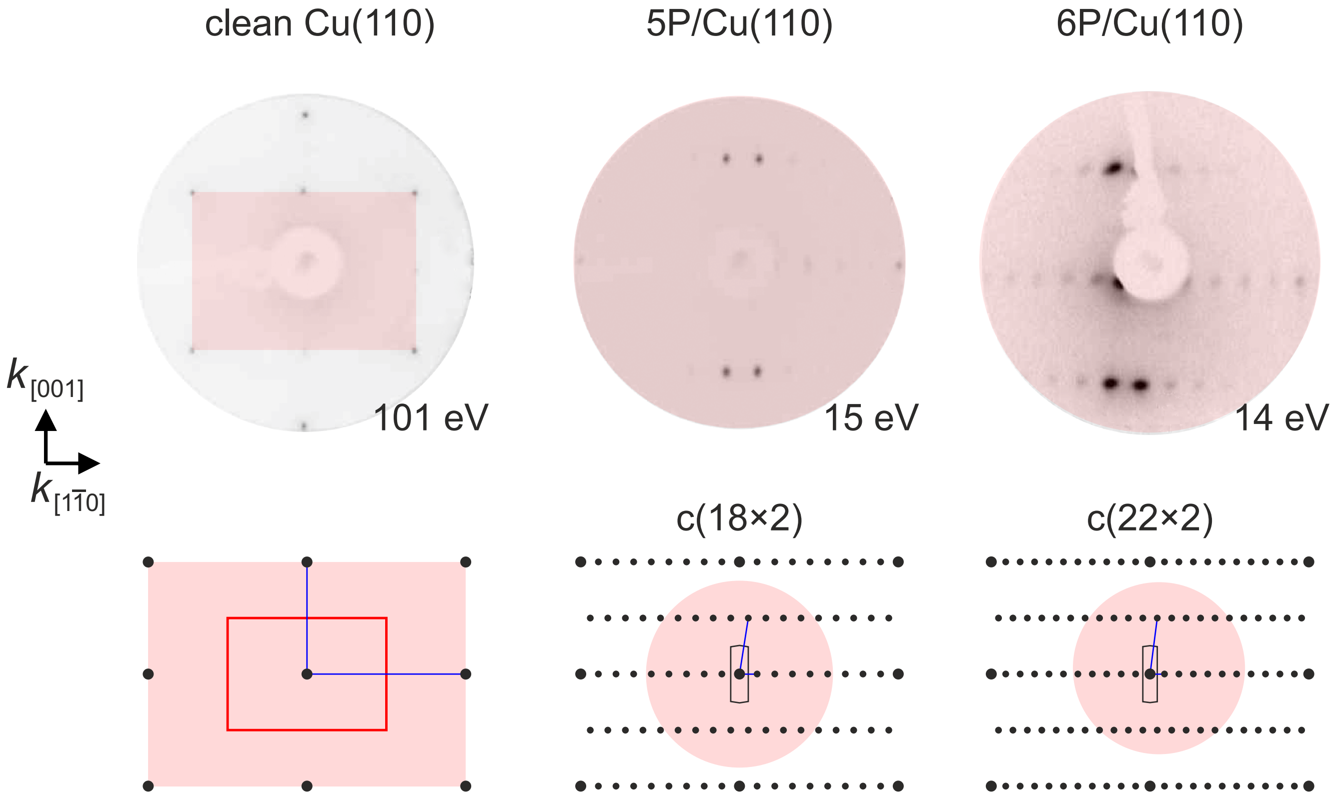

Supplementary Figure 1. Structure of 5P/Cu(110) and 6P/Cu(110). Low energy electron diffraction (LEED) images (top row) and corresponding models (bottom row) of the clean Cu(110) surface (left) and monolayers of 5P (middle) and 6P (right) on Cu(110). In the models, reciprocal lattice points are shown as black dots, the reciprocal unit cell vectors of the superstructures as blue lines, the 1BZ of Cu(110) as a red rectangle, and the 1BZ of the molecular monolayers as black polygons. The electron energies in the measurements are given next to each LEED image. To aid the comparison between LEED images and models, corresponding momentum space regions are marked as red shaded areas.

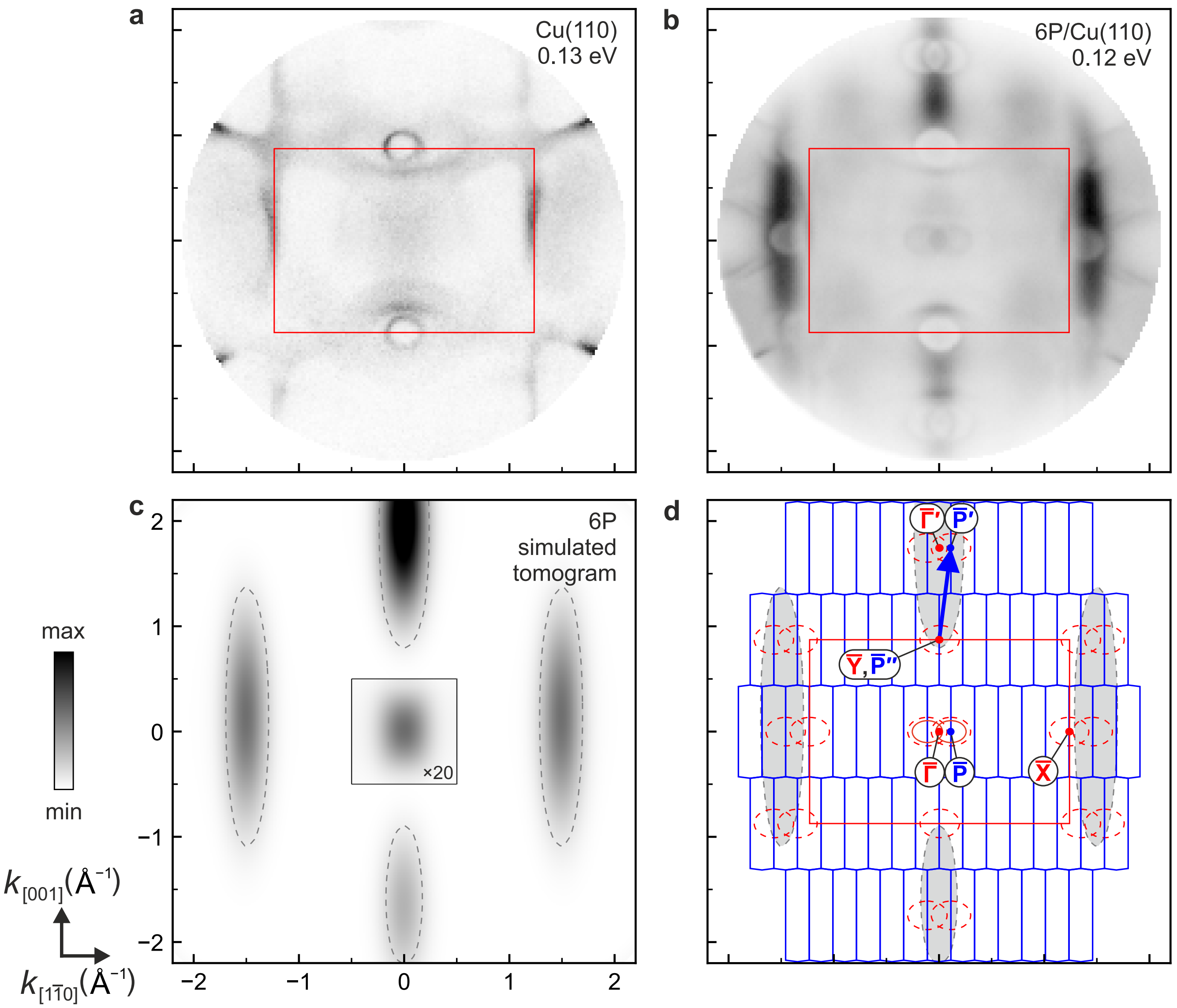

Supplementary Figure 2. Momentum maps of clean Cu(110) and para-sexiphenyl molecules (6P) on Cu(110). **a**, Experimental momentum map of clean Cu(110) at 0.13 eV binding energy. **b**, Experimental momentum map of 6P/Cu(110) at 0.12 eV binding energy. The experimental momentum maps in panels a and b correspond to $\mathbf{k}_{\|}$-resolved densities of states. **c**, Theoretically simulated tomogram of free planar 6P. Dashed lines mark the molecular emission lobes. Within the marked box centred at $\mathbf{k}_{\|} = 0$, the intensity is increased by a factor of 20. **d**, Schematic drawing illustrating the observed features in the experimental momentum map. The red rectangle shows the 1BZ of the Cu(110) surface, with high-symmetry points labeled in red. The blue polygons represent the periodically repeated first Brillouin zones of the ordered 6P/Cu(110) overlayer structures, with high-symmetry points labeled in blue. A representative reciprocal lattice vector is shown by the blue arrow. Grey dashed lines (from panel c) and grey shaded areas mark the molecular emission lobes. The red dashed ellipses mark the surface-projected bulk band gap at $\overline{\mathrm{Y}}$ and its observed replica, while the two solid red ellipses around $\overline{\mathrm{P}}$ points close to $\overline{\Gamma}$ mark the molecule-on-metal hybrid interface state (see text for more details).

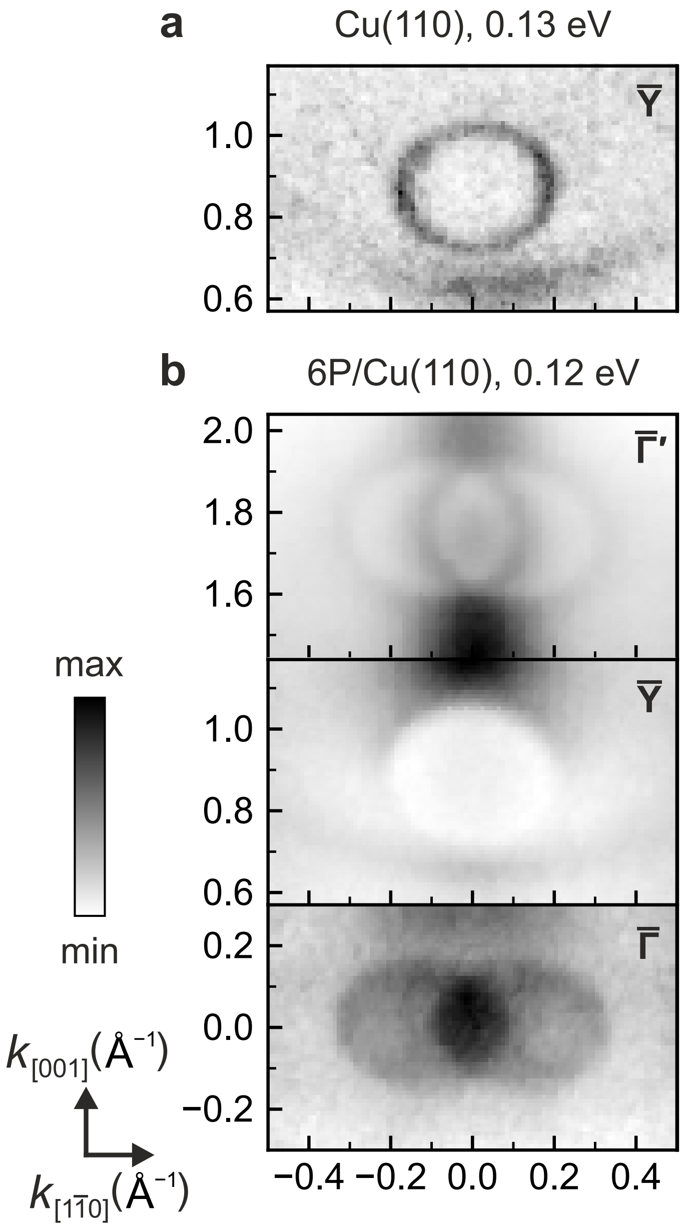

Supplementary Figure 3. Zoomed momentum maps around high-symmetry points of Cu(110). **a**, clean Cu(110) around $\overline{\mathrm{Y}}$. **b**, 6P/Cu(110) around $\overline{\Gamma}'$, $\overline{\mathrm{Y}}$, and $\overline{\Gamma}$. The different sizes of the depletion zones at $\overline{\mathrm{Y}}$ and close to $\overline{\Gamma}'$ on the one hand and the interface state close to $\overline{\Gamma}$ on the other hand can be observed clearly.

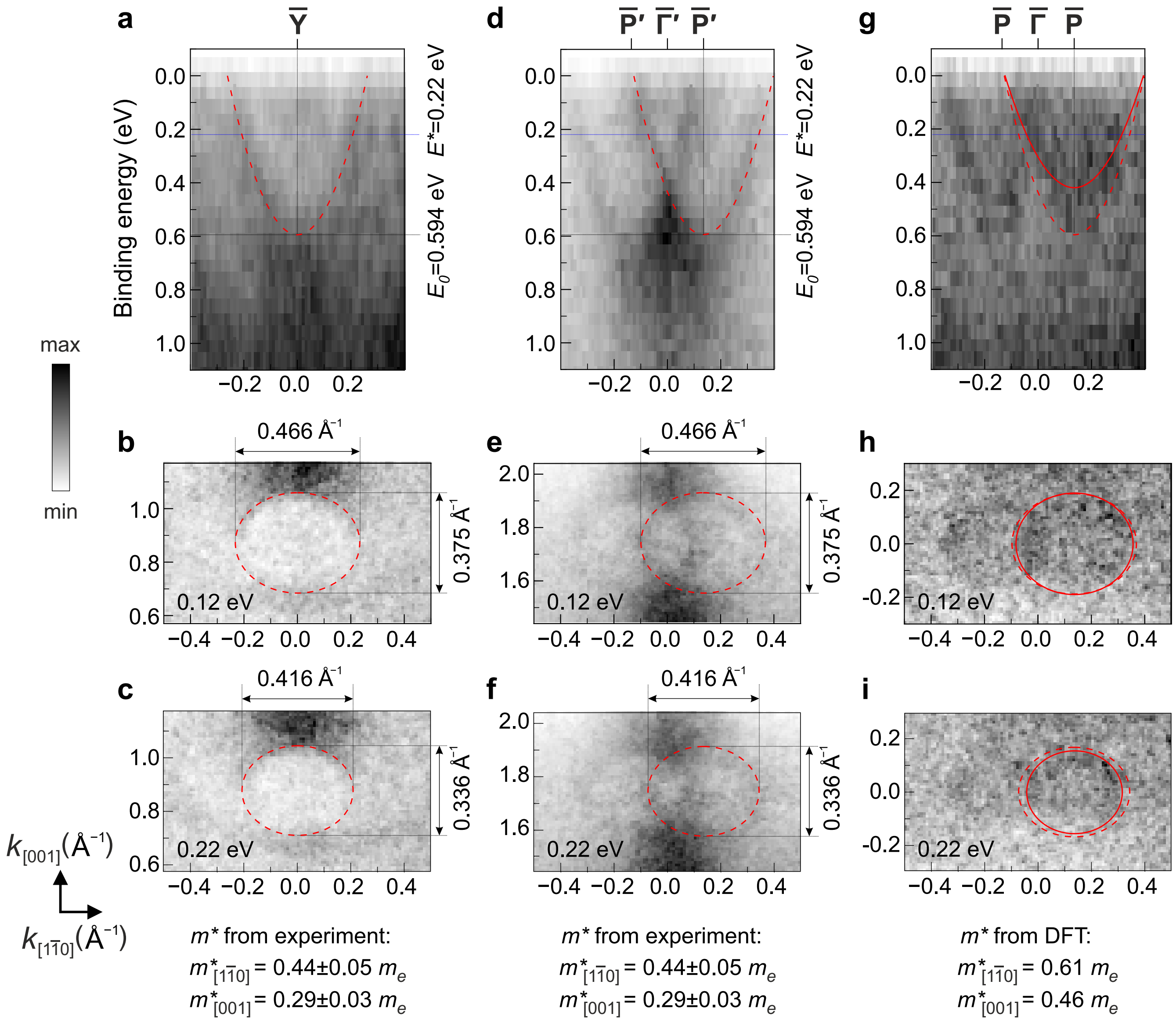

Supplementary Figure 4. Experimental band maps and corresponding momentum maps of 5P/Cu(110). Band maps and momentum maps (the latter for the binding energies of 0.12 eV and 0.22 eV), in the regions of the $\overline{\mathrm{Y}}$ point (**a**, **b**, **c**), the $\overline{\Gamma}'$ point (**d**, **e**, **f**), and the $\overline{\Gamma}$ point (**g**, **h**, **i**). The band maps are measured along the $k_{[1\bar{1}0]}$ direction of Cu(110). In panels a-i, dashed red lines and dashed red ellipses mark the edge of the surface-projected bulk band gap. In panels g-i, solid red lines and solid red ellipses mark the hybrid interface state of 5P/Cu(110). Blue horizontal lines in panels a, d, and g mark the binding energies E^* of the corresponding momentum maps in panels c, f, and i, which have been used to calculate the effective masses. Black horizontal lines in panels a, d, and g mark the apex energies E_0 of the red dashed parabolas. Vertical and horizontal lines in b, c, e, and f mark the $\mathbf{k}_{\|}$-space dimensions $\Delta k_{\|}$ of the dashed red ellipses. The effective masses are calculated as $m^* = \hbar^2(\Delta k_{\|}/2)^2/(2(E_0 - E^*))$ in the two principal directions $k_{[1\bar{1}0]}$ and $k_{[001]}$.

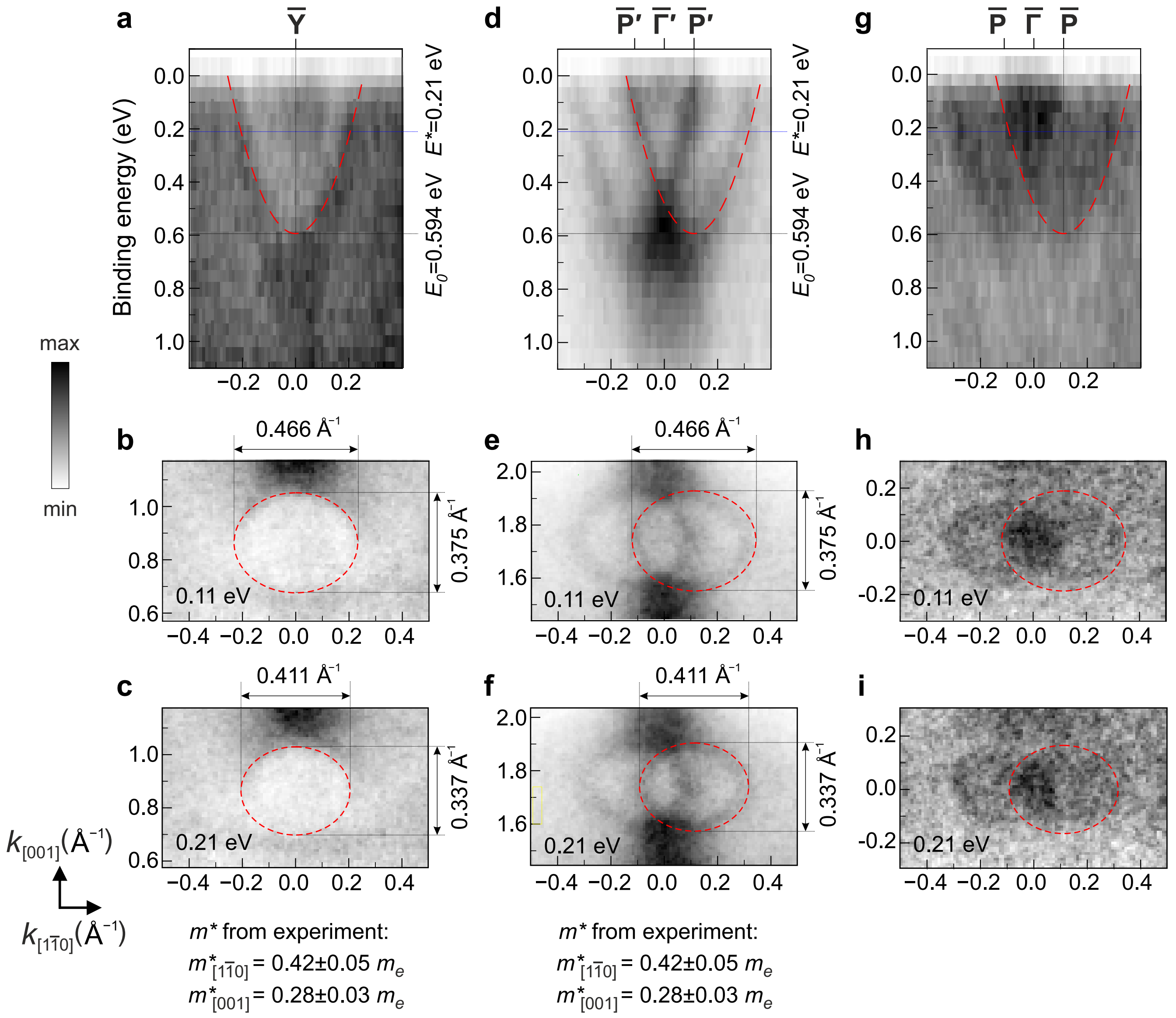

Supplementary Figure 5. Experimental band maps and zoomed momentum maps of 6P/Cu(110). Band maps and momentum maps (the latter for the binding energies of 0.11 eV and 0.21 eV), in the regions of the $\overline{\mathrm{Y}}$ point (**a**, **b**, **c**), the $\overline{\Gamma}'$ point (**d**, **e**, **f**), and the $\overline{\Gamma}$ point (**g**, **h**, **i**). The band maps are measured along the $k_{[1\bar{1}0]}$ direction of Cu(110). In panels a-i, dashed red lines and dashed red ellipses mark the edge of the surface-projected bulk band gap. Blue horizontal lines in panels a, d, and g mark the binding energies E^* of the corresponding momentum maps in panels c, f, and i. Black horizontal lines in panels a, d, and g mark the apex energies E_0 of the red parabolas. Vertical and horizontal lines in b, c, e, and f mark the $\mathbf{k}_{\parallel}$-space dimensions $\Delta k_{\parallel}$ of the dashed red ellipses. The effective masses are calculated as $m^* = \hbar^2(\Delta k_{\parallel}/2)^2/(2(E_0 - E^*))$ in the two principal directions $k_{[1\bar{1}0]}$ and $k_{[001]}$.

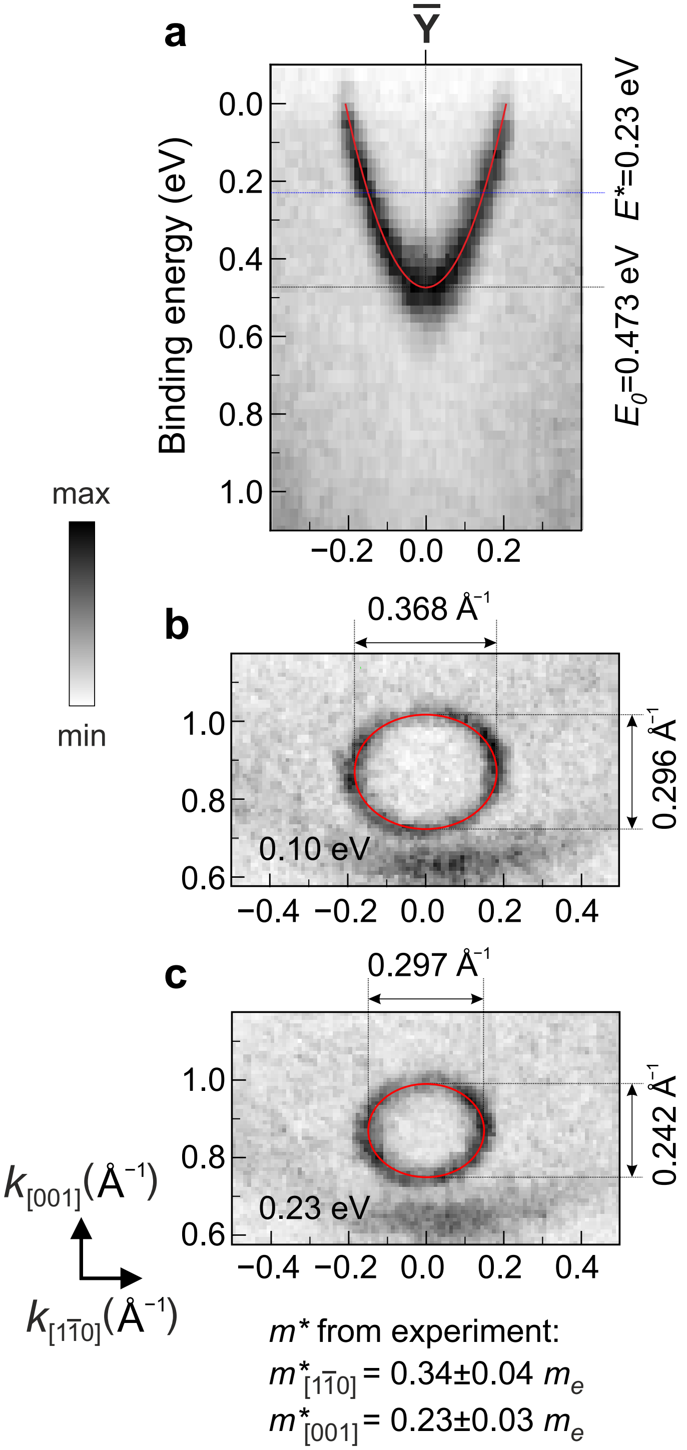

Supplementary Figure 6. Experimental band map and momentum map of clean Cu(110). Band map (**a**) and momentum maps (**b**, **c**) of the Cu(110) surface state, the latter for the binding energies of 0.10 eV and 0.23 eV) in the region of the $\bar{\text{Y}}$ point. The band maps are measured along the $k_{[1\bar{1}0]}$ direction of Cu(110). The blue horizontal line in panel a marks the binding energy E^* of the corresponding momentum map in panel c. The black horizontal line in panel a marks the apex energy E_0 of the red parabola. Vertical and horizontal lines in panel b and c mark the $\mathbf{k}_{\|}$-space dimensions $\Delta k_{\|}$ of the red ellipses. The effective mass is calculated as $m^* = \hbar^2(\Delta k_{\|}/2)^2/(2(E_0 - E^*))$ in the two principal directions $k_{[1\bar{1}0]}$ and $k_{[001]}$.

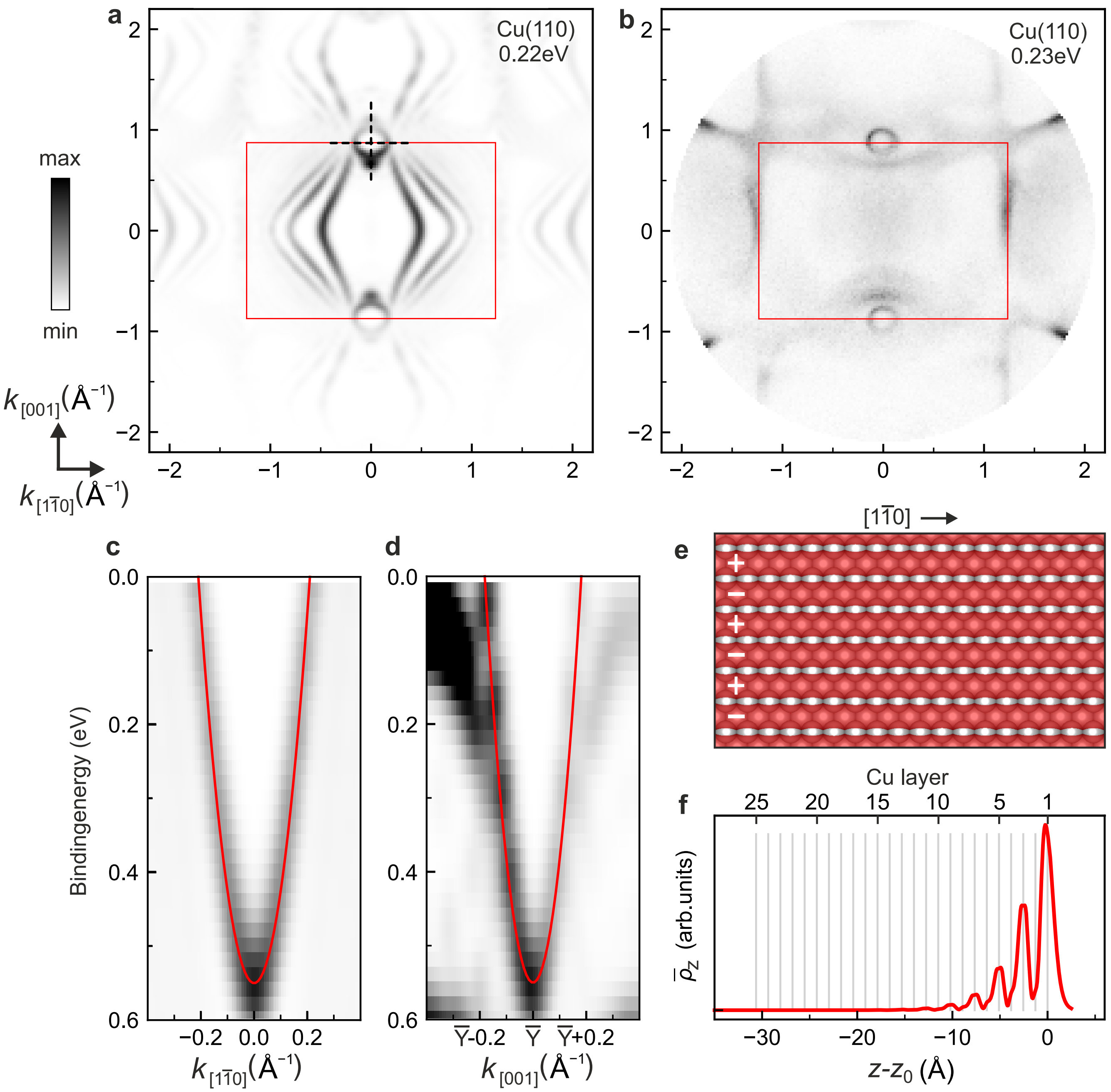

Supplementary Figure 7. Comparison of simulated and experimental momentum maps of clean Cu(110). a, Theoretical momentum map, based on a DFT calculation for the Cu(110) surface and simulated as a tomogram according to Eq. 1, but with an exponentially damped plane wave as final state. **b**, Measured momentum map of clean Cu(110), reproduced from Fig. 2a for ease of comparison with panel a. The red rectangles in panels a and b show the 1BZ of the Cu(110) surface. **c, d**, Simulated band maps along two high-symmetry directions of Cu(110), plotted for $\mathbf{k}_{\|}$ values along the horizontal and vertical dashed lines in panel a. Red solid lines mark the parabolically dispersing surface state of Cu(110). **e**, Top view of the electron density distribution of the Cu(110) surface state at $\bar{Y}$, superimposed on the atomic model of the Cu(110) surface. The sign of the surface state wave function is marked in white. **f**, Side view of the same electron density, but plane-averaged parallel to the slab surfaces and plotted across the slab that was used for the Cu(110) calculations. Solid grey lines denote the positions of atomic layers of copper.

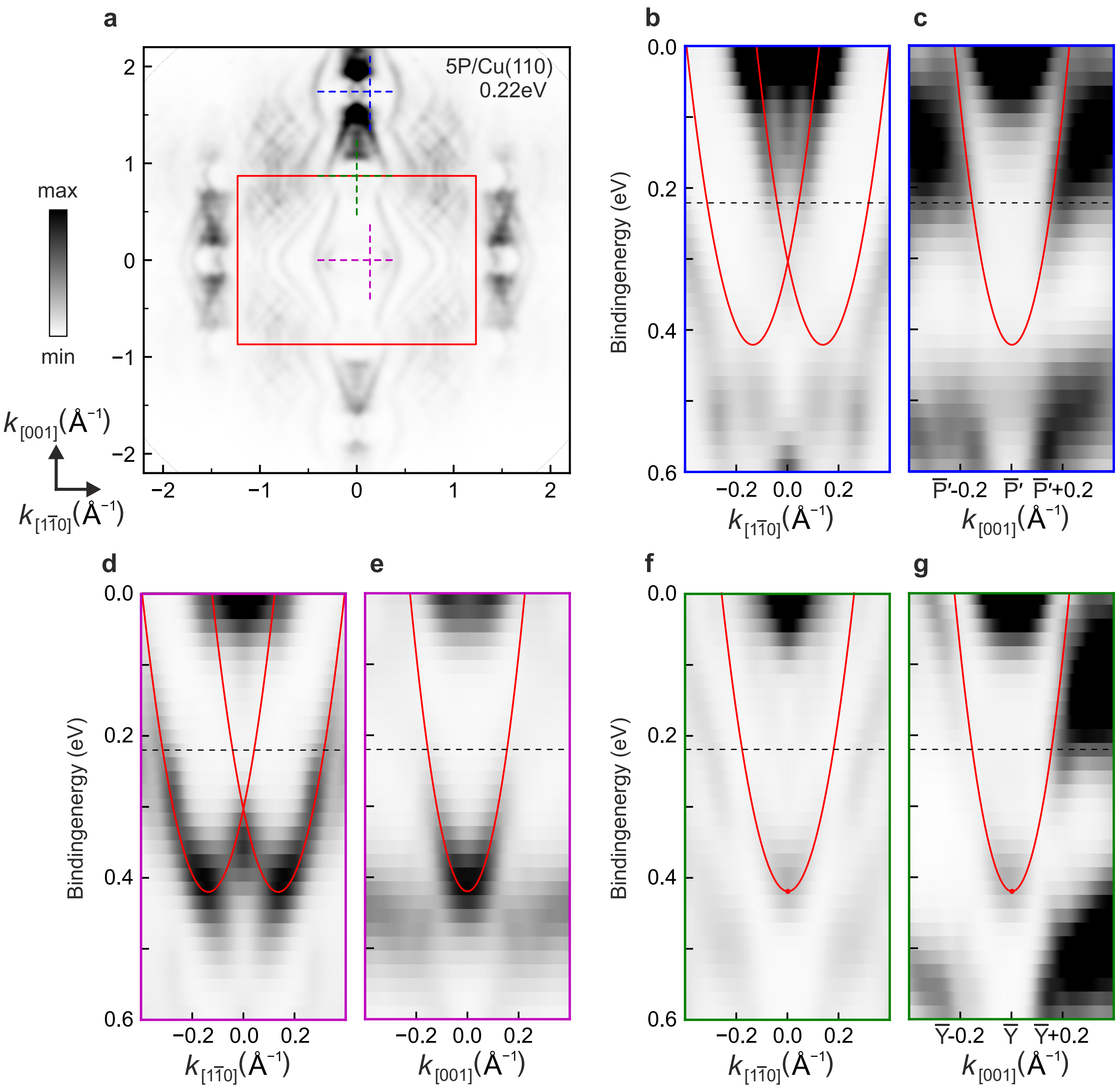

Supplementary Figure 8. Simulated momentum map and band maps of 5P/Cu(110). **a**, Theoretical momentum map, based on a DFT calculation for the 5P/Cu(110) interface and simulated as a tomogram according to Eq. 1, but with an exponentially damped plane wave as final state. The red rectangle shows the 1BZ of the Cu(110) surface. **b, c**, Simulated band maps along two high-symmetry directions of Cu(110), for $\mathbf{k}_{\|}$ values along the blue horizontal and vertical dashed lines in panel a. **d, e**, Simulated band maps along two high-symmetry directions of Cu(110), for $\mathbf{k}_{\|}$ values along the red horizontal and vertical dashed lines in panel a. **f, g**, Simulated band maps along two high-symmetry directions of Cu(110), for $\mathbf{k}_{\|}$ values along the green horizontal and vertical dashed lines in panel a. Red solid lines mark the parabolically dispersing 5P/Cu(110) interface state fitted to the band maps in panel d and e. The interface state appears weakly in panels f and g, but not at all in panels b and c.
