# Supplementary material for: Momentum-selective orbital hybridisation
Source: Nat Commun. 2022 Sep 2;13:5148. doi: 10.1038/s41467-022-32643-z (PMC9440066; doi:10.1038/s41467-022-32643-z)
Supplement: Supplementary file 2 — Description of Additional Supplementary Files [file 41467_2022_32643_MOESM2_ESM.pdf]

File Name: Supplementary Movie 1

Description: Sequence of simulated momentum maps for para-quinquephenyl on Cu(110), in the binding energy range from 0 to 0.6 eV.
